# Development and validation of a person-centered abortion scale: the experiences of care in private facilities in Kenya

**DOI:** 10.1186/s12905-020-01071-w

**Published:** 2020-09-19

**Authors:** May Sudhinaraset, Amanda Landrian, Patience A. Afulani, Beth Phillips, Nadia Diamond-Smith, Sun Cotter

**Affiliations:** 1 Community Health Sciences, University of California, Los Angeles, Jonathan and Karin Fielding School of Public Health, 650 Charles E Young Dr. S, Los Angeles, CA USA; 2 Institute for Global Health Sciences, University of California, San Francisco, School of Medicine, 550 16th Street, San Francisco, CA USA

**Keywords:** Abortion, Quality of care, Person-centered care, Kenya, Patient experience, Patient-provider communication

## Abstract

**Background:**

There is a need for a standardized way to measure person-centered care for abortion. This study developed and validated a measure of person-centered abortion care.

**Methods:**

Items for person-centered abortion care were developed from literature reviews, expert review, and cognitive interviews, and administered with 371 women who received a safe abortion service from private health clinics in Nairobi, Kenya. Exploratory factor analyses were performed and stratified by surgical abortion procedures and medication abortion. Bivariate linear regressions assessed for criterion validity.

**Results:**

We developed a 24-item unifying scale for person-centered abortion care including two sub-scales. The two sub-scales identified were: 1) Respectful and Supportive Care (14 items for medication abortion, 15 items for surgical abortion); and 2) Communication and Autonomy (9 items for both medication and surgical abortion). The person-centered abortion care scale had high content, construct, criterion validity, and reliability.

**Conclusions:**

This validated scale will facilitate measurement and further research to better understand women’s experiences during abortion care and to improve the quality of women’s overall reproductive health experiences to improve health outcomes.

## Background

Positive patient experience is a critical component in ensuring quality abortion care for women, linked to health outcomes such as decreases in severe abortion complications and seeking timely care [1]. Globally, there are approximately 56 million abortions yearly [2]. In Kenya, changes to the Constitution were passed in 2010, allowing abortions in instances where the health or the life of the mother is at risk [3]. However, the Kenya law is still restrictive and requires a qualified health professional to determine if the mother’s health or life is jeopardized. These restrictions continue to limit women’s access to quality care – resulting in abortion providers working in contexts where women experience high levels of fear and uncertainty, mistreatment and discrimination, and consequently women delaying timely care. Given these circumstances, there is evidence of high maternal mortality as a result of unsafe abortions in Kenya [4]. Safe abortion services can help prevent these avoidable deaths, and such services are available even in places like Kenya where laws are restrictive [5]. Women’s experiences during these services could help inform women’s decisions to seek safe abortions or delay abortion care, particularly for post-abortion complications [6]. Improving women’s experiences within broader efforts to improve quality of abortion services is critical in protecting women’s reproductive rights and needs.

One overlooked aspect of quality of abortion care is person-centered care, or care that is respectful of and responsive to women’s preferences, needs and values [1]. A recent review of person-centered care for abortion services found many instances where women are treated poorly [7], particularly in contexts with restrictive laws. In Brazil, for example, women report discrimination from providers, threats from health facility staff to involve law enforcement, lack of pain management provision, and long waiting lines at the facility [8]. Treating women with respect and dignity during abortion care is important from a human rights perspective, but also because women’s experiences can impact outcomes, adherence to post-abortion guidance, and future health-seeking decisions by the patient and women who hear of her experiences [7, 9].

While there is evidence that women experience poor person-centered abortion care around the globe, a significant limitation in the literature is the lack of a standardized scale to assess person-centered abortion care (PCAC). In Kenya, there are few studies that focus on patient experiences of care, with most studies qualitative in nature [4, 10–12]. Standardized measures are important for quality improvement efforts, designing and evaluating health system and policy improvements, identifying the unmet need of the most vulnerable populations, and for advocacy. A recent systematic review maps out measures of quality of abortion services [13], grouping these under structure, process, output, and outcomes. The broad range of indicators evidenced in this review demonstrates that abortion care quality is multidimensional and emphasizes the need for progress in the field to move beyond a clinical-only perspective [13]. As the authors also highlight, a streamlined list of standard indicators to measure abortion quality must be produced to advance program, advocacy, and policy efforts.

This study aims to develop and validate a PCAC scale to assess women’s person-centered experiences during abortion care in Kenya. This is the first study to validate a person-centered abortion care scale. We describe the scale development process and procedures to assess content, construct, and criterion validity. Given the unique dimension of abortion care, we examine person-centered care items separately for medication abortions and surgical abortions, acknowledging the important differences in these methods and women’s subsequent abortion experiences.

## Methods

### Scale development

**Fig. 1:**
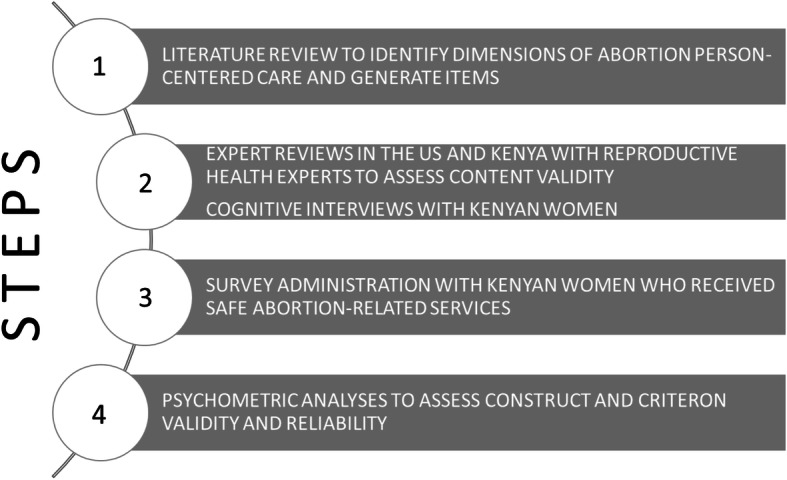
Flowchart of research methodology for the development and validation of a person-centered abortion care scale

Study activities included a literature review to identify potential items of the scale, expert reviews and cognitive interviews, survey administration with Kenyan women, and psychometric analyses to assess construct and criterion validity (Fig. 1). This study followed a standard sequential procedure to scale development to explore women’s experiences of abortion care [14] including: 1) identifying dimensions of PCAC and item generation; 2) conducting expert reviews and cognitive interviews; 3) survey administration; and 4) psychometric analyses.

This study occurred concurrently with the validation of person-centered maternity care (PCMC) scale [15] and person-centered family planning care (PCFP) scale [16], as part of a study on person-centered reproductive health care. Specifically, steps 1–3 occurred simultaneously, and step one informed a framework for equity in person-centered reproductive health care [9].

### Step 1: dimension of abortion person-centered care and item generation

This study first identified dimensions, or domains, of abortion person-centered care focused on quality of care for abortion described in detail in another manuscript [9]. Using this literature review, we identified eight domains discussed in full elsewhere [9] and which are shown in Table 1. Using the domains as a guide and based on existing measures drawn from the literature review, we then created a database of potential person-centered care measures for abortion services. In total, we began with 62 items that spanned all domains identified.

**Table 1:** Items for person-centered abortion care scale in Kenya

Original Domain	Question	Referred to in text as:	Comment
Dignity/Respect	How did you feel about the amount of time you waited?	Time to care	Retained for both
Dignity/Respect	During your time in this clinic did the doctors, nurses, or other health care providers introduce themselves to you when they first came to see you?	Introduce self	Retained for both
Dignity/Respect	Did the doctors, nurses, or other health care providers call you by your name?	Called by name	Retained for both
Dignity/Respect	Did the doctors, nurses, or other staff at the facility treat you with respect?	Treated with respect	Retained for both
Dignity/Respect	Did the doctors, nurses, and other staff at the facility treat you in a friendly manner?	Friendly	Retained for both
Dignity/Respect	During your time in the health facility, would you say you were treated differently because of any personal attribute … like your age, marital status, number of children, your education, wealth, your connections with the facility, or something like that?	Treated differently	Retained for both
Dignity/Respect	Did the doctors, nurses, and other staff at the facility show that they cared about you?	Cared	Retained for both
Dignity/Respect	Did you feel the doctors, nurses, or other health providers shouted at you, scolded, insulted, threatened, or talked to you rudely?	Verbal abuse	Retained for both
Dignity/Respect	Did you feel like you were treated roughly like pushed, beaten, slapped, pinched, physically restrained, or gagged?	Physical abuse	Retained for both
Privacy/Confidentiality	When you were speaking to the doctors, nurses or other staff at the facility, did you feel other people not involved in your care could hear what you were discussing?	Privacy	Retained for both
Privacy/Confidentiality	Do you feel like your health information was or will be kept confidential at this facility?	Record confidentiality	Retained for both
Autonomy	Did you feel like the doctors, nurses or other staff at the facility involved you in decisions about your abortion care?	Involvement in care	Retained for both
Autonomy	Did the doctors, nurses or other staff at the facility ask your permission/consent before doing procedures on you?	Consent before procedures	Retained for both
Communication	Did the doctors, nurses or other staff at the facility speak to you in a language you could understand?	Language understand	Deleted for both
Communication	Did the doctors and nurses explain to you why they were doing examinations or procedures on you?	Explain exams	Retained for both
Communication	Did the doctors and nurses explain to you why they were giving you any medicine, including pain medicine or medicine to start an abortion?	Explain medicines	Retained for both
Communication	Did you feel you could ask the doctors, nurses or other staff at the facility any questions you had?	Ask questions	Retained for both
Supportive Care	Did the doctors and nurses at the facility talk to you about how you were feeling?	Ask about feeling	Retained for both
Supportive Care	Did the doctors and nurses ask how much pain you were in?	Ask about pain	Retained for both
Supportive Care	Do you feel the doctors or nurses did everything they could to help control your pain?	Pain medication given	Retained among SA sub-group; Deleted among MA sub-group
Supportive Care	Did you feel the doctors and nurses paid attention to you during your stay in the facility?	Paid attention	Retained for both
Supportive Care	During your time at the facility, did any staff at the facility ask you or your family for *kitu kidogo [unofficial cost]*?	Bribe	Deleted for both
Supportive Care	Do you think there was enough health staff in the facility to care for you?	Enough staff	Retained for both
Supportive Care	Did you feel the doctors, nurses or other staff at the facility took the best care of you?	Took best care	Retained for both
Supportive Care	Did you feel you could completely trust the doctors, nurses or other staff at the facility with regards to your care?	Trust	Retained for both
Supportive Care	In general, did you feel safe in the health facility?	Safe	Retained for both

Adapted from Afulani et al.,[15]

### Step 2: expert review and cognitive interviews

We conducted expert reviews, in both the United States and Kenya, with reproductive health experts. The purpose of the expert reviews was to assess content validity – the extent to which measures fully describe and represent a construct (i.e. person-centered care) [14]. Additionally, we sent the surveys to individual experts focused on person-centered care for specific feedback, including differences in experiences of women for medication vs. surgical abortion. Expert reviewers gave feedback on the list of measures, suggested measures to include, and commented on item wording and response options. Expert reviewers also suggested including the same questions for both surgical and medication abortions to be able to compare across the two procedures, and because the preparation for medication abortion clients (i.e. ultrasounds, tests, etc.) would warrant the same type of questions as surgical procedures.

Based on these revisions, we conducted cognitive interviews, using an interview guide provided in Additional file 1, with 15 participants in Nairobi, Kenya in 2017. We recruited women aged 15–24 in the clinic after they had received any type of safe abortion service from one of four clinics. The purpose of cognitive interviews was to assess how important questions are to women, potential problems with survey questions, whether questions are interpreted as intended, whether the questions are contextually appropriate or needed editing, and whether the survey length was appropriate [14]. Interviews were individually conducted, coded, and analyzed by a team of three researchers (specifically, authors MS, NDS, and PA).

### Step 3: survey administration

We piloted the full survey with 30 items among a convenience sample of clients (*n* = 31) in three private clinics in Nairobi (Table 1). Based on pilot results, several survey questions were removed to accommodate time constraints and avoid interviewee fatigue. Four items were removed from the initial set of items (facility/bathrooms are clean, the facility is not crowded, electricity, and water are available in facility). The final set of 26 items was administered in the full survey as provided in Additional file 2.

Inclusion criteria for the survey were women who: 1) received a safe abortion-related service at the clinic the day of recruitment (surgical abortion or medication abortion), 2) were at least 18 years old, 3) spoke English or Swahili, and 4) owned a mobile phone where she felt comfortable being contacted by the study team. The study enumerator obtained written consent prior to any study procedures from interested and eligible women. Consented women were then administered a 40-min baseline questionnaire at the clinic in a private space. In total, 371 women were interviewed at six clinics in Nairobi. Participants received airtime equivalent to approximately $1.50 as a token of appreciation for their participation.

### Step 4: psychometric analyses

Psychometric analyses were conducted to assess construct and criterion validity and reliability. While expert reviewers believed all items would pertain to both medication abortion and surgical abortion procedures, we hypothesized that the extent to which certain behaviors would pertain more for medication abortion vs. surgical abortion procedures might differ. Therefore, all analyses were stratified by abortion type (medication vs. surgical). The following psychometric analyses were performed for both groups.

First, we examined the distribution of each item for heterogeneity. Negative items were reverse coded so that all items could be scored from worst (score of 0 on the item) to best (score of 3 on the item). Responses of “not applicable” were recoded to the highest response category (corresponding to highest PCAC) to obtain a uniform scale for conducting psychometric analyses. After recoding items, a correlation matrix was constructed and the correlations among the items were examined.

Next, we conducted exploratory factor analysis (EFA) using principal factoring. The degree of common variance among items was assessed with the Kaiser-Meyer-Olkin (KMO) test using a standard cutoff of 0.50 indicating that items are sufficiently correlated to warrant principal components analysis [17]. We then began the initial EFA by examining a scree plot of eigenvalues using all 26 scale items to determine the number of factors to retain. We used both Kaiser’s rule of retaining only factors with eigenvalues greater than one and the shape of the plot [18], along with theoretical considerations, to decide on a final number of factors. Multiple rounds of factor analyses were subsequently performed, whereby a factor loading cutoff of 0.10 was used for determining which items to delete or retain. Each factor was assessed individually before evaluating the final scale on a single factor.

We used oblique rotation because it allows for naturally occurring correlation between the rotated factors [19]. Internal consistency of the full scale and each sub-scale was then assessed using Cronbach’s alpha. Cronbach’s alpha may range from 0 to 1, with a higher value implying greater reliability. An alpha of at least 0.70 is generally considered to suggest acceptable reliability [19].

Finally, criterion validity was assessed by examining whether the PCAC scale was related to other measures of perceived quality of care. This was done by first summing the responses across all items to obtain a total PCAC score, as well as total scores for each sub-scale, where higher scores indicate better PCAC. We then ran bivariate linear regression analyses to assess the association between PCAC score (for the full scale and each sub-scale) and receipt of adequate information regarding the abortion procedure as a measure of quality of care. Women were considered to have received adequate information regarding their abortion procedure if they reported to have been told the following: about the care to be received so they knew what to expect; what side effects to expect; the warning signs to look out for that would warrant returning to the clinic or nearest hospital; and that they would be able to get pregnant again quickly, even before their next menstruation. There was a measure of satisfaction of care; however, there was little heterogeneity in distribution of the responses, with more than 95% of women indicating that they were satisfied with their abortion care. We therefore used another measure of women’s quality of care. All analyses were performed using StataSE version 15.

## Results

### Demographic characteristics

A total of 353 women completed all PCAC scale items; their demographic characteristics are presented in Table 2, stratified by abortion procedure type. Among both surgical abortion and medication abortion participants, most women were aged 20–29 years (63 and 68%, respectively) and not married, partnered, or cohabitating (73 and 81%, respectively). About half of women in both samples had a college or university degree (52% in the surgical abortion sample and 49% in the medication abortion sample). Slightly more women were employed for pay among the surgical abortion sample (62%) compared to the medication abortion sample (55%). A higher proportion of women among the medication abortion sample (54%) reported this to be their first pregnancy than in the surgical abortion sample (40%).

**Table 2:** Demographic characteristics stratified by abortion type

***Characteristic***	***N*** (%)	
	Surgical Abortion (***N*** = 157)	Medication Abortion (***N*** = 196)
Age, years		
Less than 20	6 (3.8)	25 (13.8)
20–24	61 (38.9)	76 (38.8)
25–29	37 (23.6)	57 (29.1)
30–34	25 (15.9)	25 (12.8)
35 or older	28 (17.8)	13 (6.6)
Married, partnered, or cohabitating		
Yes	42 (26.8)	37 (18.9)
No	114 (72.6)	158 (80.6)
Missing	1 (0.6)	1 (0.5)
Education		
Primary or less	20 (12.7)	14 (7.1)
Secondary or vocational	56 (35.7)	87 (44.4)
College or University	81 (51.6)	95 (48.5)
Employed for pay		
Yes	97 (61.8)	108 (55.1)
No	60 (38.2)	88 (44.9)
Religion		
Catholic, Protestant, or other Christian	148 (94.3)	189 (96.4)
Muslim	5 (3.2)	5 (2.6)
None	3 (1.9)	2 (1.0)
Missing	1 (0.6)	0 (0.0)
Number of pregnancies		
1	63 (40.1)	105 (53.6)
2	41 (26.1)	46 (23.5)
3	25 (15.9)	22 (11.1)
4	11 (7.0)	17 (8.7)
5 or more	17 (10.8)	6 (3.1)
Number of births		
0	76 (48.4)	120 (61.2)
1	37 (23.6)	40 (20.4)
2	23 (14.7)	25 (12.8)
3 or more	21 (13.4)	11 (5.6)
Number of children		
0	78 (49.7)	121 (61.7)
1	35 (22.3)	43 (21.9)
2	24 (15.3)	20 (10.2)
3 or more	20 (12.7)	12 (6.1)
Received adequate information regarding abortion procedure		
No	88 (56.1)	75 (38.3)
Yes	69 (43.9)	121 (61.7)

### Exploratory factor analysis

Among the medication abortion sample, nearly 50% of women responded “not applicable” to the “pain medication given” item. Further, only one woman responded “yes” to the “bribe” item among the surgical abortion sample and all the women in the medication abortion sample responded “no, never.” As a result, the “bribe” item was removed from both samples and the “pain medication given” item was removed among the medication abortion sample. This left 25 scale items among the surgical abortion sample and 24 items among the medication abortion sample at the start of conducting factor analyses. All scale items among both samples had a KMO measure of sampling adequacy greater than 0.50, with an overall KMO value of 0.74 among the surgical abortion sample and 0.80 among the medication abortion sample, providing evidence that the items were sufficiently correlated for conducting principal components analysis.

#### Surgical abortion sample

Among the surgical abortion sample, the initial EFA yielded a 3-factor solution with eigenvalues of greater than 1, accounting for about 50% of the variance. All 25 items loaded onto at least one of the three factors at the 0.10 factor loading cutoff, with cross loading on 11 items. We therefore categorized the items that cross loaded to factors based on the factor they loaded higher on and theoretical reasoning. Of the 11 items that loaded positively onto more than one factor, eight were retained in the factor they loaded highest on. Two items, “explain exams” and “ask questions,” which had slightly higher factor loadings on Factor 1 than Factor 2, were categorized into Factor 2 for conceptual reasons. Similarly, the item “pain medication given” had a higher loading on Factor 2 but was ultimately categorized with Factor 1. Also, despite the item “language understand” having a positive loading of greater than 0.10, upon further review it was felt that the wording of the item was too ambiguous, and as a result, the item was removed. Thus, this process resulted in 12 items being categorized to Factor 1, 9 items being categorized to Factor 2, and only three items (“treated differently,” “verbal abuse,” and “physical abuse”) remaining in Factor 3. Upon further discussion, we decided three items were insufficient for a sub-scale, and given that these items were conceptually related to those included in Factor 1, they were categorized with the items in Factor 1.

We then performed EFA with oblique rotation again on the two factors, or sub-scales, separately (Table 3). For the first factor, a standardized alpha of 0.78 was obtained suggesting acceptable reliability. Eleven of the 15 items had factor loadings of at least 0.30 and two items had factor loadings of at least 0.10. The remaining two items, “privacy” and “physical abuse,” had factor loadings less than 0.10 but were retained due to their theoretical importance. For the second factor, a standardized alpha of 0.69 was obtained. With the exception of “called by name” and “involvement in care,” the remaining seven items had factor loadings greater than 0.30. The standardized alpha for the nine items was 0.72 suggesting acceptable reliability.

**Table 3:** Rotated factor loadings of sub-scales stratified by abortion type

***Item***	Rotated Factor Loadings			
	Surgical Abortion (24 items total)		Medication Abortion (23 items total)	
	Respectful and Supportive Care (15 items)	Communication and Autonomy (9 items)	Respectful and Supportive Care (14 items)	Communication and Autonomy (9 items)
Time to care	0.3208		0.2312	
Treated with respect	0.5565		0.6147	
Friendly	0.4865		0.6244	
Treated differently	0.1974		0.4978	
Cared	0.5379		0.5557	
Privacy	0.0613^a^		0.2580	
Record confidentiality	0.1548		0.3769	
Pain medication given	0.5453		NA	
Paid attention	0.8119		0.6811	
Verbal abuse	0.4189		0.6824	
Physical abuse	0.0539^a^		0.4982	
Enough staff	0.6007		0.2242	
Took best care	0.8304		0.7268	
Trust	0.4935		0.6571	
Safe	0.5398		0.4339	
Introduce self		0.4468		0.4611
Called by name		0.2496		0.2876
Involvement in care		0.2069		0.4135
Consent before procedures		0.6446		0.3996
Explain exams		0.4508		0.3301
Explain medicines		0.5950		0.1952
Ask about feeling		0.6432		0.7358
Ask questions		0.4473		0.2669
Ask about pain		0.6197		0.6138

Notes: *NA* Not applicable as this item was deleted among medication abortion patients

^a^Factor loading is less than 0.10 but item is being retained due to theoretical importance

A final factor analysis was conducted on the remaining 24 items restricted to a single factor (Table 4). Twenty-one of the 24 items loaded onto the single dominant factor at the 0.10 cutoff, and in fact, 17 of these had factor loadings greater than 0.30. The item “called by name” had a factor loading less than 0.10; however, it was ultimately retained because of its acceptable factor loading on the sub-scale (Factor 2). “Privacy” and “physical abuse” items also had factor loadings less than 0.10, as in the two-factor solution, but again, were retained due to their theoretical importance – lack of patient privacy and physical abuse are considered central components of poor person-centered care, and thus, would be important to measure. The standardized alpha of the 24-item scale was 0.82 (mean score = 61.84; SD = 7.69; Range = 25–71). A summary of standardized alphas and associated means, standard deviations (SD), and the range of scores for the full scale and each sub-scale are provided in Table 5.

**Table 4:** Full PCAC scale rotated factor loadings stratified by abortion type

Item	Rotated Factor Loading	
	Surgical Abortion	Medication Abortion
Time to care	0.2977	0.2349
Treated with respect	0.5408	0.6111
Friendly	0.4508	0.6248
Treated differently	0.2419	0.4521
Cared	0.5051	0.5736
Privacy	0.0447^a^	0.2038
Record confidentiality	0.1710	0.4074
Pain medication given	0.6478	NA
Paid attention	0.7917	0.7092
Verbal abuse	0.3945	0.6321
Physical abuse	0.0035^a^	0.4484
Enough staff	0.5660	0.2288
Took best care	0.7686	0.7301
Trust	0.4592	0.6596
Safe	0.4708	0.4115
Introduce self	0.3128	0.2845
Called by name	0.0299^a^	0.2789
Involvement in care	0.1147	0.2810
Consent before procedures	0.5083	0.3673
Explain exams	0.5021	0.1683
Explain medicines	0.4877	0.2647
Ask about feeling	0.5783	0.2860
Ask questions	0.5160	0.4295
Ask about pain	0.5334	0.1539

Notes: *NA* Not applicable as this item was deleted among medication abortion patients

^a^Factor loading is less than 0.10 but item is being retained due to theoretical importance

**Table 5:** Standardized alphas and means for the PCAC scale and sub-domains stratified by abortion type

	Alpha (standardized)	Mean Score	SD	Min	Max
**Surgical Abortion**					
Full PCAC Scale (24 items)	0.82	61.84	7.69	25	71
Respectful and Supportive Care (15 items)	0.78	41.13	4.17	20	45
Communication and Autonomy (9 items)	0.72	20.71	4.79	4	27
**Medication Abortion**					
Full PCAC Scale (23 items)	0.82	56.98	7.44	13	69
Respectful and Supportive Care (14 items)	0.82	38.16	4.10	6	42
Communication and Autonomy (9 items)	0.65	18.82	4.98	7	27

Note: *SD* Standard deviation

#### Medication abortion sample

Among the medication abortion sample, the initial EFA using principal factors with oblique rotation also yielded a 3-factor solution with eigenvalues of greater than 1, accounting for about 53% of the variance. All 24 items loaded onto at least one of the three factors at the 0.10 factor loading cutoff, with cross loading on 12 items. We categorized the items that cross loaded to factors based on the factor they loaded higher on and theoretical reasoning. Ten of the 12 items that loaded positively onto more than one factor were categorized according to the higher factor loading. Two items, “friendly” and “privacy”, had similar loadings on two factors but were categorized with Factor 1 because these items were deemed to be more conceptually related to the items in that factor. Despite “explain medicines” loading to Factor 1, it was ultimately recategorized to Factor 3 for conceptual reasons. As was done in the surgical abortion sample, the “language understand” item was removed after it was concluded that the item was too ambiguous. This process resulted in 12 items being categorized to Factor 1, three items (“treated differently,” “verbal abuse,” and “physical abuse”) being categorized to Factor 2, and 9 items being categorized to Factor 3. For consistency, the three items in Factor 2 were ultimately categorized with Factor 1 to yield a final 2-factor solution, like that proposed among the surgical abortion sample, with 23 items retained at this time.

Performing EFA with oblique rotation on the two factors, or sub-scales, separately, we found that the first factor had a standardized alpha of 0.82 suggesting acceptable reliability. All 14 items had factor loadings greater than 0.10, and 11 of the 14 items had factor loadings greater than 0.30 (Table 3). For the second factor, a standardized alpha of 0.65 was obtained. All items in Factor 2 had factor loadings greater than the 0.10 cutoff; six of the nine items had factor loadings greater than 0.30.

A final factor analysis was conducted on the remaining 23 items restricted to a single factor (Table 4), with all items loading onto the single factor at the 0.10 cutoff. The standardized alpha of the final 23 item scale was 0.82 (mean score = 56.98; SD = 7.44; Range = 13–69; Table 5).

To name the factors, we assessed the specific items and mapped the items out on the original domains that the authors conceptualized for person-centered care. For the first factor, items came from the domains of respectful and supportive care (RSC sub-scale). Items included in the second factor all related to communication and women’s ability to be involved in care (CA sub-scale). Therefore, again, guided by the original conceptualized domains of person-centered care, we named the second factor “Communication and Autonomy.”

### Criterion validity

The results of the bivariate linear regressions assessing the association between the two PCAC sub-scales (RSC sub-scale and CA sub-scale) and the full PCAC scale and the receipt of adequate information regarding the abortion procedure, respectively, are provided in Table 6. Among both samples, we found that women who reported receiving adequate information regarding their abortion procedure had significantly higher PCAC scores (for each sub-scale, as well as the full scale) than women who did not receive adequate information. These results confirm our hypothesis and suggest that higher PCAC scores are associated with receiving adequate information.

**Table 6:** Bivariate linear regression of person-centered abortion care sub-scales and full-scale and receipt of adequate abortion procedure information stratified by abortion type

	RSC Sub-scale	CA Sub-scale	PCAC Full Scale
**Surgical Abortion**			
Received adequate information regarding abortion procedure			
No	Ref	Ref	Ref
Yes	2.01 (0.72, 3.30)**	4.07 (2.68, 5.45)***	6.06 (3.82, 8.33)***
Constant	40.25 (39.39, 41.11)***	18.92 (18.00, 19.84)***	59.17 (57.68, 60.66)***
R-squared	0.0515	0.1730	0.1494
**Medication Abortion**			
Received adequate information regarding abortion procedure			
No	Ref	Ref	Ref
Yes	1.44 (0.27, 2.62)*	1.68 (0.25, 3.10)*	3.12 (1.00, 5.23)**
Constant	37.27 (36.35, 38.19)***	17.79 (16.66, 18.91)***	55.05 (53.39, 56.72)***
R-squared	0.0245	0.0219	0.0368

Notes: Coefficient (95% confidence interval) provided. *RSC* Respectful and Supportive Care. *CA* Communication and Autonomy. PCAC=Person Centered Abortion Care

*p < 0.05; **p < 0.01; ***p < 0.001

## Discussion

This study utilized established scale development procedures to validate a new scale for PCAC. The resulting 24- and 23-item scale for surgical abortion and medication abortions, respectively, includes two sub-scales that measure: 1) Respectful and Supportive Care (i.e. whether women received care in a respectful manner, trusted providers, and felt safe in the facility); and 2) Communication and Autonomy (i.e. whether women were actively involved in making healthcare decisions and providers fully explained procedures, exams, treatment regimens, etc. in a way a woman could understand beforehand). The scale has high content, construct, and criterion validity, as well as strong reliability, based on the expert reviews, cognitive interviews, and psychometric analyses. The few existing related scales are either much more narrowly focused, for example measuring only one component of person-centered care related to stigma [20], or are much broader, including the quality of technical aspects of the abortion itself [13]. Our scale measures a diverse set of domains of person-centered care, while still being focused on the quality of women’s experiences themselves.

This study examines the potential for customized scale items for medication abortion versus surgical abortion procedures, and we found significant overlap in items across the two methods. While surgical procedures can be completed in less than 15 min, the preparation and wait times at the health facility and, potentially, at home is typically longer for those receiving a medication abortion. Consequently, the interactions with the provider are likely to be different. However, the one minor difference included a question regarding pain, which was not included in the person-centered scale for medication abortions. This minor difference suggests that a unified scale can be applicable for both medication abortion and surgical abortion clients, with an additional pain question asked for surgical clients only. Given that this same scale could potentially be used for all abortion clients regardless of abortion type, this scale is more generalizable and user-friendly for clinics and potentially scalable if integrated into other larger, on-going surveys. Additionally, many researchers and others consider the two types of abortion services to be vastly different; these findings suggest a convergence in this field that the same set of factors are equally important to women’s person-centered abortion experiences, regardless of method.

The two identified sub-scales (RSC sub-scale and CA sub-scale) are in line with other measures of person-centered care for reproductive health, specifically PCMC [15] and PCFP [16], but also diverged in a couple areas. For example, similar to PCMC, autonomy and communication domains produced one sub-scale for PCAC. On the other hand, for PCFP, autonomy was more closely associated with items for respectful care. Additionally, in the respectful care domain, similar to PCMC, physical and verbal abuse were included in the abortion care scale, but not PCPF scale, although in both cases the item on physical abuse was retained based on theoretical rationale. Potential reasons for these similarities in maternity and abortion scales include that, similar to maternity care, provision of abortion procedures, particularly surgical abortions, requires greater attention and time from providers than providing contraceptive services. Therefore, the longer and more in-depth the provider-patient interaction is may naturally lead to more opportunities for certain behaviors to occur during care. Nonetheless, the similarity of items across PCMC, PCFP, and PCAC scales suggest that the construction and manifestation of person-centered care may be similar across maternity care, family planning, and abortion care. Future studies may be able to use items found across the three scales to examine whether there are differences in experiences across reproductive health care services.

There were a few limitations to the PCAC scale validation for abortion. One limitation is that we recruited women who received an abortion-related service from a private clinic/hospital. The extent to which these measures hold up to other settings, including public hospitals where women may experience substandard care, is unknown. Recruiting in private clinics may have biased our sample. For example, the prevalence of women with a college or university degree is high and may be due to recruitment in private facilities, where the quality of care may be higher, and thus attract higher socioeconomic status women. Future studies may be conducted among women accessing abortion services from the public sector. Our study also includes women seeking abortions in legal settings and it is difficult to ascertain its applicability in settings where laws are even more restrictive than Kenya. Likely, these women would potentially face higher levels of poor person-centered care and experience additional issues captured by items that were dropped from our validation process, such as bribes. Another limitation is that due to constraints in the duration of the surveys, we were unable to include items related to the health facility environment. We anticipated that because private non-governmental organization clinics tend to have better health facility environments than public facilities, that this might play less of a role in our study sample, but is likely to be more important in public facilities. In addition, given that the surveys were conducted within the health facility, social desirability bias in responses is a limitation despite emphasis on confidentiality and privacy during the consent process.

## Conclusion

We developed and validated a new scale for PCAC. This scale makes an important contribution to the literature given growing evidence of the importance of prioritizing person-centered care across reproductive health services and the lack of validated measurement tools. There are a number of potential uses of this scale. First, a validated scale for PCAC will facilitate measurement and research to better understand women’s experiences when seeking care for abortion. Second, it will facilitate future research to identify the gaps in PCAC during women’s abortion care experiences. This scale will also aid research into sources of disparities in women’s abortion care experiences and help refine interventions aiming to improve abortion care, particularly for vulnerable groups. In addition, it provides a valuable tool for evaluating these types of interventions in other contexts. The scale will also facilitate standardized measurement across key places where person-centered care is most lacking during abortion care and simultaneously assess trends in the provision of abortion care. Future studies may assess how person-centered abortion care may influence or correlate with different outcomes, including abortion complications or post-abortion family planning counseling. Finally, given the similarities in items across the PCMC, PCFP, and PCAC scales, together these reproductive health scales could facilitate future research on person-centered care across the reproductive health continuum and improve women’s reproductive health experiences and outcomes worldwide. This scale is potentially applicable in other settings beyond Kenya. Validation in other settings is however needed to assess its validity across settings.

## Supplementary information

**Additional file 1.**

**Additional file 2.**

## Data Availability

The datasets analyzed during the current study are available from the corresponding author upon reasonable request.

## Abbreviations

CA: Communication and Autonomy
EFA: Exploratory factor analysis
KMO: Kaiser-Meyer-Olkin
PCAC: Person-centered abortion care
PCFP: Person-centered family planning care
PCMC: Person-centered maternity care
RSC: Respectful and Supportive Care
