# Supplementary material for: Development and validation of a person-centered abortion scale: the experiences of care in private facilities in Kenya
Source: BMC Womens Health. 2020 Sep 19;20:208. doi: 10.1186/s12905-020-01071-w (PMC7501655; doi:10.1186/s12905-020-01071-w)
Supplement: Supplementary file 1 — Additional file 1. [file 12905_2020_1071_MOESM1_ESM.docx]

**Start time: ____:____**

*Audio recording introduction:* This is *NAME* on *DATE*. I am with respondent *RESP ID.* Thank you for giving me permission to record this interview.

**Extended Cognitive Interview Guide**

**(SPARQ Kenya only)**

**Semi-Structured Interview Guide: Safe Abortion / Post-Abortion Care**

1. Tell me about what health-related service you received at the facility today (eg. Safe abortion – medical or surgical or post abortion care)?
2. Can you tell me about your experience leading up to seeking this service today?
3. How did you find out that you were pregnant?
4. Have you ever used a family planning method? Tell me about it.
5. Can you describe what you know about family planning methods?
   - 1. *Probe*: How knowledgeable do you feel about family planning?
     2. *Probe*: Would you want to know more?
6. Tell me about what you know about your menstrual cycle?
   - 1. *Probe*: Can you describe what you know about menstruation and how it relates to getting pregnant?
7. How did you hear about the health services at this facility?

a. Tell me about any other place in the area that provides similar services?

i. What made you choose Marie Stopes and not any of the other facilities?

b.   Where do you go to get abortion-related information (e.g. internet, healthcare provider, friend or family member)?

c.     How do you like to receive information about your health (e.g. brochure/ pamphlet, letter, e-mail, SMS, social media)?

1. I’d like to talk you about the quality of care you received at the health facility today. How do you feel about the quality of care you received at the health facility today?
   1. Tell me about how the staff at the facility explained your procedure/ care to you?
      1. Did you find it easy to understand? Why?
   2. Tell me about whether the staff at the facility allowed you to express your concerns about your care in your own words?
      1. How did they do this?
   3. Tell me about how welcoming or unwelcoming the staff were.
      1. Tell me about what they did to make you feel this way.
   4. Tell me about whether you trust that the information you shared with the facility will be kept private.
      1. Why do you feel this way?
   5. How did you feel about the space you were provided for your counseling and for your procedure?
      1. Tell me about whether you felt no one else could see or hear you.
   6. Tell me about the amount of information the staff gave you about the pain you might experience from the procedure?
      1. Tell me about whether you felt it was enough or not enough. Why?
   7. Tell me about whether any of the staff members yelled at you/ hit you/ made you feel bad about yourself at any point during your care today?
      1. How did you feel about that?
2. Tell me about any factors that make it difficult for women to seek healthcare services?
   1. Tell me about any religious groups or communities whose beliefs or practices make it difficult to seek healthcare services?
      1. How do those religious groups/ communities discourage women from seeking healthcare services?
      2. What about any community norms/practices?
      3. (If client says none,) how would you feel if a religious group/ community did something to make it difficult for you to seek healthcare? What makes you say that?
      4. How does stigma influence women's ability to seek healthcare services?
      5. How do cultural beliefs and tradition influence women's ability to seek healthcare services?
3. How does your community and/or religion feel about abortion or post-abortion care?
   1. Tell me about whether your community/religious thinks abortion is always wrong?
      1. Can you explain why they might feel that way?
   2. In your community and/or religion, what do people think about women who have abortions (eg. considered selfish, seen as sinners, rejected by family and/or friends)?
      1. Why do you think they feel that way?
4. Based on how your community/religion feels about abortion, tell me about whether you are worried about how others will perceive you after you leave the health facility today.
   1. Tell me about whether you are worried…
      1. That other people might find out about your abortion?
         1. What makes you feel that way?
      2. That you will disappoint someone you love?
         1. What makes you feel that way?
      3. That people will gossip about you?
         1. What makes you feel that way?
      4. That you will be rejected by someone you love?
         1. What makes you feel that way?
      5. That people will judge you negatively for your decision?
         1. What makes you feel that way?
5. In general, tell me about whether you feel supported by people that you are close to about having an abortion/receiving PAC?
   1. Tell me about whether someone came with you to the facility for your procedure?
      1. *If someone came with her to the facility*: Tell me about whether the provider asked if you wanted that person to join you during the procedure?
      2. *If not:* Tell me about whether you would have liked someone to join you during the procedure. Why?
   2. Tell me about whether you have someone who can care for you when you return home after the procedure?
      1. *If yes:* Who?
      2. *If no:* Would you like someone to care for you at home after the procedure?
   3. Tell me about whether you feel that you can talk to people that you are close with about the care you received.
      1. Who can you talk to?
      2. Tell me about to what extent you can talk to them.
6. Tell me about whether you believe that your age or marital status influenced the quality of care you received today.
   1. Tell me whether anyone at this health facility told you that you were too young to receive services?
      1. Can you tell me what happened?
      2. How did this make you feel? / How would you feel if this happened?
   2. Tell me about whether anyone at this facility told you that you were required to get permission from your parents or husband/partner for the services you received today?
      1. Can you tell me what happened?
      2. How did this make you feel? / How would you feel if this happened?
   3. Tell me about whether you feel that you were treated differently from other clients in this facility because you are unmarried?
      1. Can you tell me what happened?
      2. How did this make you feel? / How would you feel if this happened?
   4. Tell me about whether you feel like the services at this facility are available to both unmarried and married women?
      1. Can you tell me what happened?
      2. How did this make you feel? / / How would you feel if this happened?
7. We have come to the end my questions. Do you have anything more you want to tell me before we finish?

**End time: ____:____**
